# Effects of 6PPD-Quinone on Human Liver Cell Lines as Revealed with Cell Viability Assay and Metabolomics Analysis

**DOI:** 10.3390/toxics12060389

**Published:** 2024-05-26

**Authors:** Yunqing Qi, Aiqing Qiu, Xinyue Wei, Yiting Huang, Qing Huang, Wei Huang

**Affiliations:** Guangdong Key Laboratory of Environmental Pollution and Health, College of Environment and Climate, Jinan University, Guangzhou 510632, China

**Keywords:** 6PPD-Q, human liver cells, cytotoxicity, transformation products, metabolomics

## Abstract

*N*-(1,3-Dimethyl butyl)-*N*′-phenyl-phenylenediamine-quinone (6PPD-Q) is a derivative of the widely used rubber tire antioxidant 6PPD, which was first found to be acutely toxic to coho salmon. Subsequent studies showed that 6PPD-Q had species-specific acute toxicity in fishes and potential hepatotoxicity in mice. In addition, 6PPD-Q has been reported in human urine, demonstrating the potential widespread exposure of humans to this chemical. However, whether 6PPD-Q poses a higher risk to humans than its parent compound, 6PPD, and could cause adverse effects in humans is still unclear. In this study, we utilized two human liver cell models (the human proto-hepatocyte model L02 and the human hepatocellular carcinoma cell line HepG2) to investigate the potentially differential effects of these two chemicals. Cell viability curve analysis showed that 6PPD-Q had lower IC_50_ values than 6PPD for both liver cell lines, suggesting higher toxicity of 6PPD-Q to human liver cells than 6PPD. In addition, L02 cells are more sensitive to 6PPD-Q exposure, which might be derived from its weaker metabolic transformation of 6PPD-Q, since significantly lower levels of phase I and phase II metabolites were detected in 6PPD-Q-exposed L02 cell culture medium. Furthermore, pathway analysis showed that 6PPD-Q exposure induced changes in phenylalanine, tyrosine, and tryptophan biosynthesis and tyrosine metabolism pathways in L02 cells, which might be the mechanism underlying its liver cell toxicity. Gene expression analysis revealed that exposure to 6PPD-Q induced excessive ROS production in L02 cells. Our results further supported the higher risk of 6PPD-Q than 6PPD and provided insights for understanding the effects of 6PPD-Q on human health.

## 1. Introduction

*N*-(1,3-dimethylbutyl)-*N′*-phenyl-p-phenylenediamine (6PPD) is widely used as an antioxidant in the rubber industry, which raised significant concerns due to its ozonized transformation product, 6PPD-quinone (6PPD-Q), identified to be of acute toxicity on several aquatic species at environmentally relevant concentrations in recent years [1]. Since the hydrolysis half-life of 6PPD-Q is significantly longer than that of 6PPD [2], subsequent studies reported the detection of 6PPD-Q in various environmental matrices, such as dust, soil, water, sediments, and atmospheric particles [3,4]. The widespread occurrence of 6PPD-Q in the environmental matrices has raised concerns about its effects on environmental and human health. Especially, the finding of 6PPD-Q in the atmosphere and human urine implied that humans might also be generally exposed to 6PPD-Q, which raises an urgent need for its toxicity assessment.

To date, the toxicity assessment of 6PPD-Q has mainly been conducted on aquatic species. Research has shown that 6PPD-Q can cause acute mortality in silver salmon by disrupting the blood–brain barrier or affecting the mitochondrial electron transport chain [5,6]. Additionally, a study found that both 6PPD and 6PPD-Q can enter the circulatory system and affect the nervous system of zebrafish larvae, leading to developmental and behavioral changes as well as cardiotoxicity at high concentrations [7]. As for toxicity to mammalians, knowledge is still very limited, especially for humans. Fang et al. found that 6PPD-Q oral administration caused damage to the liver tissue of mice via activated inflammation and disturbed glycolipid metabolism [8], revealing that 6PPD-Q might also pose the potential to cause adverse effects on the human liver.

However, there are still no human-relevant studies about the effects of 6PPD-Q on the liver. The liver is an important organ for maintaining normal physiological functions and plays an important role in metabolism and detoxification [9] and, therefore, might be the target organ of many xenobiotics. In vitro liver cell models have been widely used in toxicology for the screening of cytotoxic and genotoxic compounds and the determination of characteristic liver lesions and associated biochemical mechanisms induced by toxic compounds [10]. With the development of molecular toxicology, multiple omics techniques have been applied for toxicity assessment. Metabolomics is the systematic identification and quantification of all metabolites in a given biological sample to directly reflect the dynamics of metabolic levels under toxicant exposure or disease states to identify altered metabolic pathways resulting from an exposure factor or pathophysiological perturbation [11].

In the present study, using the human liver cell lines HepG2 and L02 as models, we systematically assessed the cytotoxicity of 6PPD-Q on human liver cells and investigated the potential metabolic perturbance via metabolomic profiling, aiming to illustrate the potential toxicity of this emerging chemical on humans.

## 2. Materials and Methods

### 2.1. Chemicals and Materials

The standards of 6PPD-Q and 6PPD were purchased from DR. EHRENSTORFER (>99% purity, LGC, Luckenwalde, Germany). Dimethyl sulfoxide (DMSO, >99.9%) and 4-chloro-phenylalanine (4-Cl-Phe) were purchased from Sigma-Aldrich (St. Louis, MO, USA). Dulbecco’s modified Eagle’s medium (DMEM), RPMI-1640 medium, fetal bovine serum (FBS), penicillin-streptomycin, 0.25% trypsin-EDTA, and phosphate-buffered saline (PBS) were from Gibco (Thermo Fisher Scientific, Waltham, MA, USA). Cell culture bottles and plates were bought from Corning (Corning Incorporated, Corning, NY, USA). All other chemicals used, including water (H_2_O), methanol (MeOH), acetonitrile (ACN), and isopropanol (IPA), were of high-performance liquid chromatography (HPLC)-grade (Merck & Co., Billerica, MA, USA). Human normal hepatocyte cell line L02 and human hepatocellular carcinoma cell line HepG2 were obtained from the American Type Culture Collection (ATCC, Manassas, VA, USA).

### 2.2. Cell Culture

As recommended by ATCC, HepG2 cells were cultured in DMEM supplemented with 10% FBS and 1% penicillin-streptomycin, and L02 cells were cultured in RPMI-1640 medium supplemented with 20% FBS and 1% penicillin-streptomycin. Both cell lines were maintained at 37 °C in a humidified environment with 5% carbon dioxide. The cells were passaged for three generations before inoculating into 96-well or 6-well plates for subsequent experiments. The 6PPD-Q and 6PPD standard solutions were prepared with DMSO to achieve a stock concentration of 1000 mg/L.

### 2.3. MTT Assay

The cells were initially plated into a 25 cm^2^ culture bottle and harvested via trypsin digestion at 37 °C for 5 min when reaching 80% confluence. Afterward, cells were inoculated into 96-well plates at a density of 20,000 per well and further incubated for 24 h. The medium in the 96-well plate was then replaced with the FBS-free medium containing 6PPD or 6PPD-Q at a final exposure concentrations of 1–1000 μg/L, and the medium containing 0.1% DMSO was used as a vehicle control. For each exposure concentration, six replicates were set up and cultured for 48 h. At the end of exposure, cell viability was assessed using the MTT Cell Proliferation and Cytotoxicity Assay Kit (Leagene, Beijing, China) following the manufacturer’s instructions. Briefly, the medium was carefully aspirated, 100 μL of fresh serum-free medium and 10 μL of MTT solution (5 mg/mL in PBS) were added to each well, and the incubation was continued for 4 h at 37 °C. Then, 100 μL of SDS-HCl solution (1 g of SDS dissolved in 10 mL of 0.01 M HCl) was added to each well. The plate was incubated in a 37 °C humidified chamber for 4 h followed by low-speed oscillation for 10 min on a shaking table to fully dissolve the purple formazan. Finally, the absorbance of each well was measured at 570 nm using an enzyme marker, and the results were expressed as a percentage of cell viability normalized to the control. As the viability curves were non-monotonic functions, we employed nonlinear fitting of the cell viability data using the dose–response inhibition function in GraphPad Prism software (version 9, GraphPad Software, Inc., La Jolla, CA, USA) after logarithmic transformation of the concentrations. This yielded the corresponding IC_50_ values, which were then assessed for quality via the R-squared values generated after fitting the curves.

### 2.4. Analysis of Transformation Products in Cell Culture Medium

The L02 and HepG2 cells were inoculated at 1 × 10^6^ cells/well in a six-well plate and cultured in complete medium to 60% confluence, followed by changing the medium to FBS-free medium with different 6PPD-Q concentrations (0 and 50 μg/L). Six duplicates were prepared for each treatment. After exposure for another 48 h, the medium was carefully collected. The cell culture medium (1 mL) collected from 0 and 50 μg/L 6PPD-Q exposure was spiked with 4-Cl-Phe as an internal standard and acidified with 400 µL of 10% formic acid/H_2_O (*v*/*v*), followed by the addition of 1.2 g of sodium sulfate and 0.6 g of sodium chloride. The samples were then extracted twice with 4 mL and 3 mL of ACN, respectively, and the extracts were combined, nitrogen-blown to near dryness, and re-dissolved in 200 µL of ACN/H_2_O (15:85, *v*/*v*) for analysis.

### 2.5. Sample Preparation for Metabolomics Analysis

The sample preparation for metabolomics was conducted as previously reported [12]. The L02 cells were inoculated at 1 × 10^6^ cells/well in a six-well plate and cultured in complete medium to 60% confluence, followed by changing the medium to FBS-free medium with different 6PPD-Q concentrations (0, 1, and 50 μg/L). Six duplicates were prepared for each treatment. After exposure for another 48 h, the medium was carefully removed, and the cells were rapidly washed with PBS and collected using a cell scraper. The cell pellet was quickly quenched by adding 600 μL of frozen MeOH/H_2_O (4:1, *v*/*v*), while 4-Cl-Phe was added as an internal standard. Three repetitive freeze–thaw cycles with liquid nitrogen were conducted for cell lysis. The samples were then centrifuged at 16,000× *g* for 10 min at 4 °C. Finally, 350 μL of the upper layer was collected for metabolomics analysis. The samples were vacuum-dried, redissolved in 100 μL of 50% MeOH/H_2_O (*v*/*v*), vortexed for 3 min, and then centrifuged at 16,000× *g* for 8 min at 4 °C. The supernatants were collected and subjected to LC-MS analysis. The proteins were retained and measured for data correction.

### 2.6. Instrumental Analysis

The metabolomics analysis and semi-quantification of the transformation products were conducted on an ultra-high-performance liquid chromatography system (UHPLC) coupled to an Orbitrap Exploris^TM^ 240 mass spectrometer (MS) (Thermo Scientific, Waltham, MA, USA). Five microliters of each sample were injected for analysis. Detailed chromatographic conditions and MS parameters are summarized in Table S1. Samples from the control and exposure groups were randomly analyzed, with solvent blanks and QC samples inserted every six samples. The samples were analyzed under both positive ionization and negative ionization modes.

### 2.7. Data Processing and Analysis

For metabolomics raw data, deconvolution, peak extraction, peak alignment, and compound identification were conducted using Compound Discoverer 3.3 (Thermo Scientific, Waltham, MA, USA). The parameters are set as follows: MS1 tolerance, 5 ppm; MS2 tolerance, 5 ppm; alignment model, adaptive curve; maximum shift, 2 min; intensity tolerance, 30%; S/N threshold, 3; and minimum peak height, 20,000. The adduct ions in positive ionization mode include [M + H]^+^, [M + K]^+^, [M + NH_4_]^+^, [M + H − H_2_O]^+^, [M + Na]^+^, [M + ACN + H]^+^, [M + ACN + Na]^+^, [2M + H]^+^, [2M + Na]^+^, [2M + K]^+^, [2M + NH4]^+^, [2M + ACN + H]^+^, and [M + ACN + Na]^+^. The adduct ions in negative ionization mode include [M − H]^−^, [M + Cl]^−^, [M + FA − H]^−^, [M − H_2_O − H]^−^, [2M − H]^−^, and [2M + Hac − H]^−^. The compounds were annotated with accurate mass-to-charge ratios (m/z) of the precursor ion and matching of the MS/MS profiles in accordance with the mzCloud and mzVault databases. Compounds that were annotated were manually identified as plausible compounds when at least three fragment ions were matched to compounds in the above libraries. The ultimate peak area of the identified compounds was corrected with the peak area of the internal standard (4-Cl-Phe for both the positive and negative ionization modes) and the protein amount of the corresponding sample. The online platform MetaboAnalyst 5.0 (https://www.metaboanalyst.ca/, accessed on 25 December 2023) was implemented to perform partial least squares discriminant analysis (PLS-DA). Differential metabolites between the exposure and control groups were filtered by *p*-value (<0.05) and multiplicity of fold change (FC, >1.20 or <0.83). The differential metabolites were then subjected to metabolic pathway and enrichment analyses using MetaboAnalyst 5.0 (https://www.metaboanalyst.ca/, accessed on 25 December 2023) according to the Kyoto Encyclopedia of Genes and Genomes (KEGG) pathway database (www.genome.jp/kegg/, accessed on 25 December 2023).

In processing of transformation product data, potential transformation products from the literature were confirmed with accurate MS1 mass and MS2 fragments using Freestyle software 1.5 (Thermo Scientific, Waltham, MA, USA). The relative peak area was used for comparison between the two cell lines.

### 2.8. RNA Extraction and Quantitative Reverse Transcription PCR

The cell treatment and collection were identical to that utilized in preparation of samples for metabolomics analysis. In brief, 1 × 10^6^ L02 cells/well were inoculated in six-well culture plates and cultured in a complete medium until 60% confluence was reached. Thereafter, the medium was changed to an FBS-free medium with different concentrations of 6PPD-Q (0, 1, and 50 μg/L). Six replicates were prepared for each concentration and each treatment was repeated three times. Following a further 48 h of exposure, the medium was carefully removed, the cells were rapidly washed with PBS, and they were collected using a cell scraper. Total RNA was extracted from the treated cells with TRIzol reagent (Invitrogen, Carlsbad, CA, USA) as per the supplied instructions. The concentration and purity of the RNA samples were detected with a NanoDrop spectrophotometer (Thermo Fisher Scientific, Waltham, MA, USA). The expression level of specific genes was assessed via quantitative reverse transcription PCR (RT-qPCR). cDNA was synthesized with reverse-transcribed total RNA using the Evo M-MLV RT Mix kit with gDNA clean for qPCR (Accurate Biology, Beijing, China) in a total volume of 20 μL according to the manufacturer’s instructions. All the primers were obtained from the Beijing Genomics Institute (Shenzhen, China) and the primer sequences were listed in Table S2. The gene expression level was calculated using the comparative Ct method and normalized against GAPDH.

### 2.9. Quantitative Analysis of Cellular Reactive Oxygen Species (ROS) Level

Cells were first inoculated into all-black 96-well cell culture plates (Corning, Corning, NY, USA) at a density of 20,000 cells per well and incubated for an additional 24 h. Subsequently, the medium in the 96-well plates was replaced with an FBS-free medium containing 6PPD-Q at concentrations of 0, 1, and 50 μg/L. Six replicates were set up for each exposure concentration, and the plates were incubated for 48 h. At the end of exposure, the cellular ROS level was assessed using the ROS Assay Kit (Beyotime, Shanghai, China) according to the manufacturer’s instructions. Briefly, at the end of the exposure, the solution was meticulously absorbed, 100 μL of a diluted 2′,7′-dichlorofluorescin diacetate (DCFH-DA) solution (10 μM) was added to each well, and the plates were incubated in an incubator for 20 min. This was followed by three washes with serum-free medium, which effectively removed the non-internalized DCFH-DA. Using a Multi-Function Measuring Instrument (Bio-Tek, Winooski, VT, USA), the fluorescence intensity was measured at 488 nm excitation wavelength and 525 emission wavelengths, and the results were expressed as normalized to the amount of ROS produced in the control group. Six replicates were set up for each concentration and each treatment was repeated three times.

### 2.10. Statistical Analysis

Statistical analyses of the metabolomics data were performed using GraphPad Prism version 9.0 for Windows (GraphPad Software, San Diego, CA, USA), OriginPro 2021 (OriginLab Corp., Armonk, NY, USA), and the online data processing website MetaboAnalyst 5 (http://www.metaboAnalyst.ca/, accessed on 25 December 2023). Comparisons between two groups were conducted with the Student’s t-test, while comparisons among groups were conducted with one-way ANOVAs followed by Tukey’s post hoc test. Two-sided *p* < 0.05 was thought to be of significance.

## 3. Results

### 3.1. 6PPD-Q Induced Differential Effects on Liver Cell Viability

**Figure 1:**
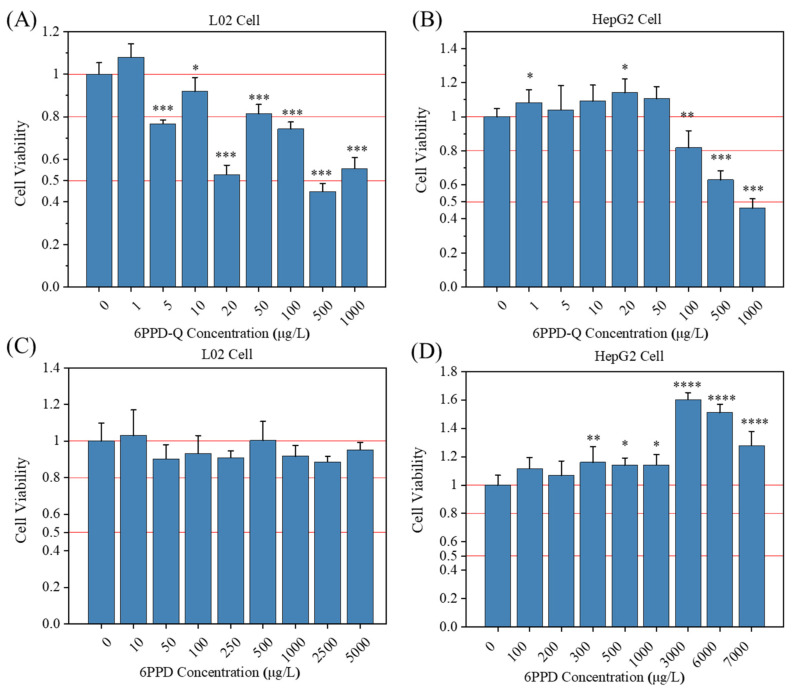
Results of cytotoxicity assay for both cells after 6PPD-Q and 6PPD exposure for 48 h. (**A**) 6PPD-Q-exposed L02 cells; (**B**) 6PPD-Q-exposed HepG2 cells; (**C**) 6PPD-exposed L02 cells; and (**D**) 6PPD-exposed HepG2 cells. Data are expressed as mean ± standard error of the mean (*n* = 6). * *p* < 0.05, ** *p* < 0.01, *** *p* < 0.001, and **** *p* < 0.0001 indicate significant differences between the exposure group and the control group.

The MTT assay revealed that after exposure to 6PPD-Q for 48 h, no significant change in cell viability was observed when the concentrations were less than 1 μg/L for both cell lines. The cell viability under 6PPD-Q exposure for both cell lines was significantly reduced when the exposure time was 48 h. However, the viability of L02 cells was significantly reduced in a dose-dependent manner when the concentration of 6PPD-Q exceeded 1 μg/L. More interestingly, we found that the L02 cell viability at exposure concentrations of 5 μg/L and 20 μg/L was lower than some of the higher exposures (Figure 1A). For HepG2 cells, a significant decrease in cell viability was observed at concentrations of 6PPD-Q exceeding 50 μg/L (Figure 1B). While for 6PPD exposure, concentrations that resulted in significant changes in cell viability were not consistent with 6PPD-Q. As shown in Figure 1C, the viability of the L02 cell did not undergo a significant decrease when exposed to any concentrations of 6PPD ranging from 0 to 5000 µg/L. For HepG2 cells, when the concentration of 6PPD was less than 300 μg/L, no significant change was observed in the cells, whereas when it was greater than 300 μg/L, the cells showed a significant increase compared with the control group (Figure 1D).

After fitting the viability curve (Figure S1), the calculated 48 h exposure IC_50_ values of 6PPD-Q for HepG2 cells and L02 cells were 127.50 µg/L and 22.51 µg/L, respectively, while the 48 h exposure IC_50_ of 6PPD exceeded the testing concentration range. These results indicated that 6PPD and 6PPD-Q induced differential liver cell toxicity while exhibiting cell line specificity, suggesting that 6PPD-Q is more toxic compared to 6PPD. Although the R-squared value derived from the IC_50_ curve fit did not demonstrate a high-quality fit, it is evident that L02 cells exhibited a heightened sensitivity to 6PPD-Q exposure.

### 3.2. Differences in the Transformation of 6PPD-Q between HepG2 and L02 Cells

**Figure 2:**
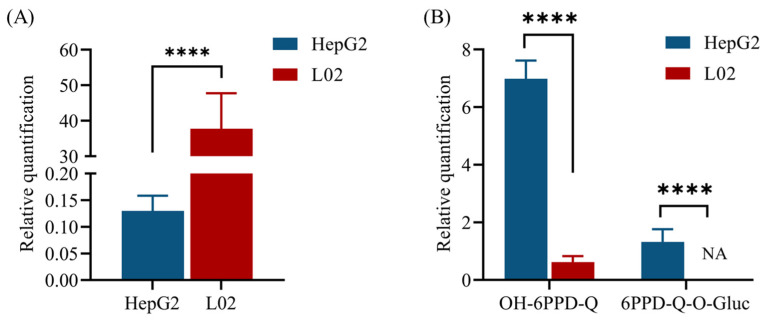
Transformation products in 50 µg/L of 6PPD-Q-exposed cell culture medium. (**A**) The residual 6PPD-Q in the two cell culture media and (**B**) 6PPD-Q biotransformation products in the two cell culture media. Data are expressed as mean ± standard error of the mean (*n* = 6). **** *p* < 0.0001 indicates significant differences between the two cell culture media. NA, not detected.

Through literature research and data analysis, two transformation products of 6PPD-Q were identified in the cell culture medium, which were its phase I metabolite hydroxylated 6PPD-Q (6PPD-Q-OH) and phase II metabolite 6PPD-Q-O-glucuronide (6PPD-Q-O-Gluc) (Table S3 and Figures S2–S4). By comparing the relative peak area of 6PPD-Q and its transformation products in the two cell culture media, we found that the residual 6PPD-Q content in the L02 cell culture medium was much higher than that in HepG2 cells (Figure 2A). The 6PPD-Q-OH content in the HepG2 cell culture medium was significantly higher than that in the L02 cell culture medium, whereas no 6PPD-Q-O-Gluc was detected in the 6PPD-Q-exposed L02 cell culture medium (Figure 2B).

### 3.3. Mechanistic Insights into the Effects of 6PPD-Q on Liver Cells

**Figure 3:**
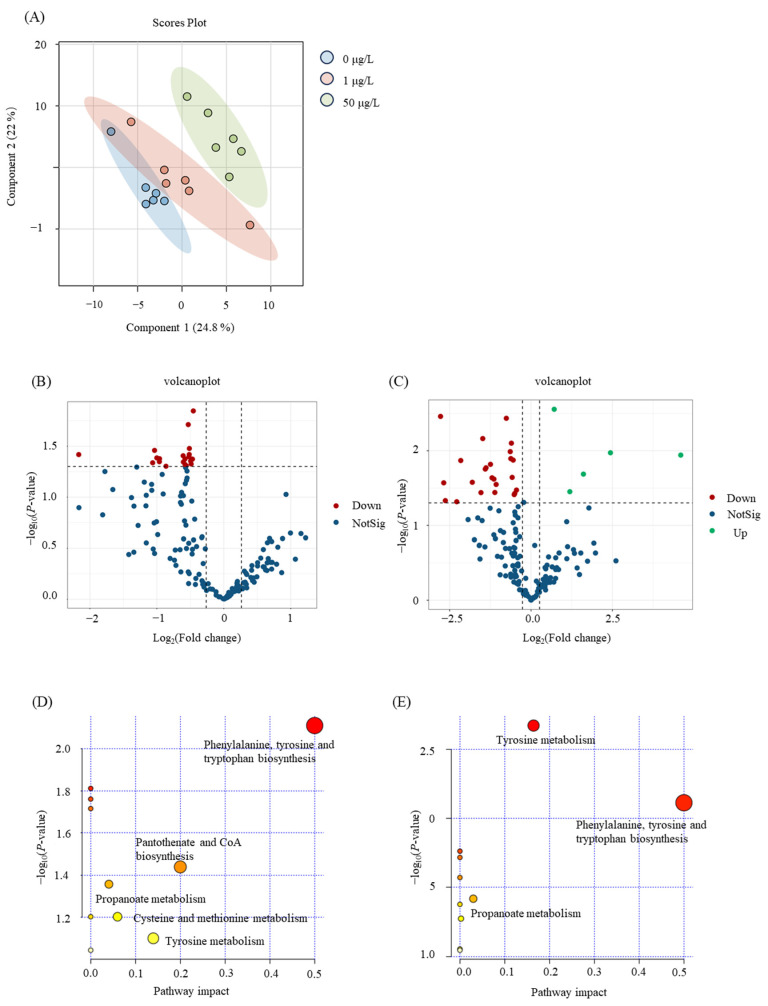
Non-targeted metabolomics analysis of the 6PPD-Q-exposed L02 cells. (**A**) PLS-DA score plots of L02 cells exposed to 6PPD-Q; (**B**) volcano plots of the metabolites between 1 μg/L of 6PPD-Q exposure and control group; (**C**) volcano plots of the metabolites between 50 μg/L of 6PPD-Q exposure and control group; (**D**) KEGG pathway analysis of the differential metabolites in 1 µg/L of 6PPD-Q-exposed L02 cells; and (**E**) KEGG pathway analysis of the differential metabolites in 50 µg/L of 6PPD-Q-exposed L02 cells.

As a newly identified chemical of concern, the pathways that underlie the effects of 6PPD-Q on human liver cells are still unknown. Since L02 cells were more sensitive to 6PPD-Q exposure, we further conducted pathway enrichment analysis with the differential metabolites in 6PPD-Q-exposed L02 cells to investigate the potential metabolic mechanism underlying the toxicity. Metabolomic analysis showed that exposure to 6PPD-Q induced metabolic perturbation in the liver cells. PLS-DA score plots showed the obvious separation of the controls from the exposure groups (Figure 3A). Using *p* < 0.05 and FC > 1.20 or <0.83 as the criteria, we found that 20 and 31 metabolites were significantly changed in L02 cells with 1 μg/L and 50 μg/L of 6PPD-Q exposure, respectively (Figure 3B,C).

Further pathway enrichment analysis results showed that the differential metabolites in the 1 μg/L exposure group were enriched in the phenylalanine, tyrosine, and tryptophan biosynthesis; pantothenic acid and CoA biosynthesis; alanine metabolism; cysteine metabolism; and tyrosine metabolism pathways (Figure 3D), while the differential metabolites of 50 μg/L were found to be enriched in the biosynthetic pathways of phenylalanine, tyrosine, and tryptophan, as well as in the biosynthesis in tyrosine metabolism and propanoate metabolism (Figure 3E). Particularly, 13 metabolites were simultaneously and consistently changed at both 1 μg/L and 50 μg/L exposure concentrations compared with the control group (Table 1), and were enriched in phenylalanine, tyrosine, and tryptophan biosynthesis and tyrosine metabolism pathways, indicating that 6PPD-Q exposure induced disruption of these amino acid metabolism pathways.

### 3.4. PPD-Q Exposure Induced Oxidative Stress on Liver Cells

**Figure 4:**
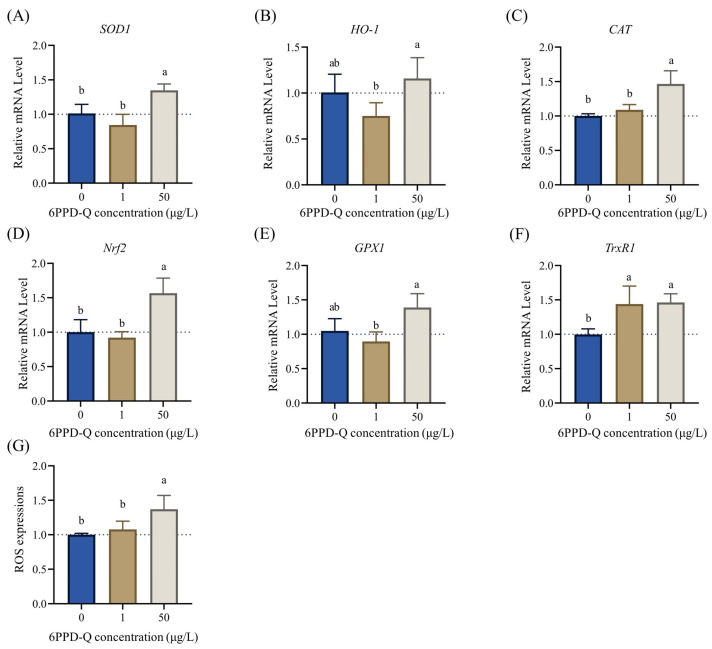
Expression of L02 cells with oxidative stress response-related genes with increasing concentration of 6PPD-Q exposure. (**A**) Superoxide dismutase (*SOD1*); (**B**) heme oxygenase-1 (*HO-1*); (**C**) catalase (*CAT*); (**D**) nuclear factor erythroid 2-related factor 2 (*Nrf2*); (**E**) glutathione peroxidase (*GPx1*); and (**F**) the mammalian selenoprotein thioredoxin reductase 1 (*TrxR1*). (**G**) Quantitative analysis of ROS expression based on DCF fluorescence intensity. Data are expressed as mean ± standard error of the mean (*n* = 6). Different letters above the columns indicate significant differences at *p* < 0.05, as determined via one-way ANOVA with Tukey’s post hoc test.

RT-qPCR analysis showed that oxidative stress-related genes were generally significantly upregulated in L02 cells after exposure to different concentrations of 6PPD-Q compared to controls. As shown in Figure 4, the expression of catalase (*CAT*), nuclear factor erythroid 2-related factor 2 (*Nrf2*), and mammalian selenoprotein thioredoxin reductase 1 (*TrxR1*) increased in a significant dose-dependent manner. The expression of superoxide dismutase (*SOD-1*), heme hydroxylase-1 (*HO-1*), and glutathione peroxidase 1 (*GPX1*) showed an inverted U-shape distribution with increasing concentration. These results demonstrated that 6PPD-Q exposure significantly induces oxidative damage responses in L02 cells. We further quantified the intracellular ROS levels. As seen in Figure 4G, the cellular ROS level exhibited a dose-dependent increase under 6PPD-Q exposure. When exposed to 50 μg/mL of 6PPD-Q, the ROS level was significantly higher than the control group. These results further indicated that 6PPD-Q exposure induced the production of cellular ROS.

## 4. Discussion

In this study, we investigated the potential toxic effects and mechanism of action of 6PPD-Q on human hepatocytes based on in vitro liver cell exposure models. The viability curve showed that the 48 h IC_50_ values of 6PPD-Q on the two human liver cell lines were significantly different, in which L02 cells were more sensitive to 6PPD-Q exposure. In addition, 6PPD-Q exhibited higher toxicity to the two liver cells in comparison with its precursor compound 6PPD. This result is consistent with that found in juvenile coho salmon, that 6PPD-Q exhibited high toxicity while 6PPD did not [1]. Zhao et al. treated mice with 6PPD and 6PPD-Q, finding that 6PPD-Q showed ~1.5–8-times higher bioaccumulation than 6PPD in all tissue types, and the liver appeared to be the major target organ for both 6PPD and 6PPD-Q after oral ingestion [13]. Di et al. found that 6PPD-Q was more stable in water solutions than 6PPD, indicating that the toxic effects of 6PPD-Q on sensitive species could be persistent in water [2]. These results also support our finding of differences in the toxicity of 6PPD and 6PPD-Q on the liver cell lines.

The viability analysis showed that L02 cells exhibited more severe cytotoxicity at low 6PPD-Q exposure compared to higher exposure, indicating that the dose–response curve is not a monotonic function, but rather a non-monotonic one. A considerable number of toxicological dose–response curves have been reported to exhibit a U-shaped, inverted U-shaped, or even a wavy pattern. As the research on 6PPD-Q is still in progress and there are still many uncharted territories awaiting investigation, we will adjust the research on 6PPD-Q toxicity at a later date to delve more deeply into the issues related to the effects produced by the 6PPD-Q dose. We also found that L02 cells are more sensitive to 6PPD-Q exposure since its 48 h IC_50_ value was lower than that of HepG2. The toxicity of 6PPD-Q to fish appears to be highly species-specific, and many of the tested fish species to date do not show any abnormal physiological responses or acute mortality, even after exposure to much greater, nonenvironmentally relevant concentrations. Montgomery et al. suggested that differences in species sensitivity to 6PPD-Q are a result of differences in basal expression of the biotransformation enzymes across various fish species since they found that tolerant species might detoxify 6PPD-Q more effectively [14]. Indeed, our semi-quantitative results of 6PPD-Q and its biotransformation products in the two cell culture media showed that HepG2 cells could metabolize 6PPD-Q into 6PPD-Q-OH (phase I) and 6PPD-Q-O-Gluc (phase II) metabolite more efficiently. Similar to fish species, this phenomenon might also arise from differences in the enzymes associated with the uptake and metabolism of 6PPD-Q in L02 and HepG2 cells. Consistent with our results, in the study of biotransformation of triclosan in L02 cells versus HepG2 cells, Zhang et al. observed that triclosan underwent a more rapid transformation in HepG2 cells [12].

The application of metabolomics further revealed that 6PPD-Q exposure affected cellular tyrosine metabolism and phenylalanine, tyrosine, and tryptophan biosynthesis in L02 cells, suggesting that 6PPD-Q may lead to liver cell toxicity by interfering with these amino acid metabolism pathways. H.C. et al. demonstrated that phenylalanine, tyrosine, and tryptophan may be involved in the pathogenesis of liver failure and hepatic encephalopathy [15]. Lower levels of tryptophan have been associated with increased metabolic inflammation and fibrosis, which may lead to non-alcoholic fatty liver disease (NAFLD) [16]. Teunis et al. have demonstrated that NAFLD-associated inflammation can be reduced by modulating tryptophan metabolism [17]. In our study, we observed that the biosynthetic pathways of phenylalanine, tyrosine, and tryptophan were disrupted in the exposure groups following 6PPD-Q exposure. It has been demonstrated that certain pollutants may cause NAFLD by affecting tyrosine metabolism [18]. The L-tyrosine content in L02 cells decreased 0.71-fold and 0.65-fold, respectively, in the 1 and 50 μg/L exposure groups. These results indicated that 6PPD-Q exposure may potentially lead to hepatotoxicity by influencing metabolic pathways such as tyrosine metabolism and tryptophan biosynthesis.

In addition, several typical oxidative stress-related genes were significantly upregulated upon 6PPD-Q exposure, suggesting that 6PPD-Q might trigger ROS production in liver cells. Previous mouse studies have shown that 6PPD-Q exposure caused partial oxidative damage and an inflammatory response in the liver of the mice [8]. In addition, Wang et al. have demonstrated that 6PPD-Q in PM_2.5_ exhibit moderate oxidative potential assessed via the acellular dithiothreitol assay [19]. The overproduction of ROS has been demonstrated to have an inescapable detrimental effect on liver function. It is well documented in the medical literature that oxidative stress plays a significant role in the pathogenesis of inflammatory chronic liver disease. Oxidative stress has been shown to trigger hepatocyte-associated emergency pathways, which in turn lead to the development of inflammation and steatosis [20]. In the same vein as above, it is well documented that ROS are involved in the formation of liver fibers leading to hyperfibrosis [21], causing liver injury [22]. Chen et al. demonstrated that antioxidants associated with oxidative stress were present in the majority of clinical samples of NAFLD, indicating a robust correlation between oxidative stress and NAFLD [23]. Therefore, our results suggested that 6PPD-Q may result in substantial damage to the human liver by inducing excessive oxidative stress.

## 5. Conclusions

In this study, the potential toxicity of 6PPD-Q on human liver cells was evaluated for the first time with in vitro cell models, finding that 6PPD-Q exhibited higher toxicity to liver cells than its precursor compound 6PPD, with differing 48 h IC_50_ values for the two human liver cell lines. HepG2 cells showed higher tolerance to 6PPD-Q exposure, which might be due to its higher metabolic detoxification capacity. Further analysis showed that 6PPD-Q may potentially lead to hepatotoxicity by inducing perturbations in phenylalanine, tyrosine, and tryptophan biosynthesis and tyrosine metabolism pathways, as well as excessive oxidative stress. This study provides basic data for future studies on the population health effects and risk assessment of 6PPD-Q.

## Figures and Tables

**Table 1:** Common differential metabolites in 1 and 50 µg/L of 6PPD-Q exposure of L02 cells ^a^.

Differential Metabolites	HMDB ID	FC ^b^ (1 μg/L vs. Control)	FC (50 μg/L vs. Control)
1-(4-Bromophenyl)-2-(7H-purin-6-ylsulfanyl) ethanol	HMDB0258663	0.73	0.67
beta-Ala-Phe	HMDB0304795	0.69	0.59
*N*-[[3-(b-D-Glucopyranosyloxy)-2,3-dihydro-2-oxo-1H-Indol-3-yl]acetyl]aspartic acid	HMDB0039409	0.70	0.71
3-[2-[[2-[[2-Acetamido-3-(4-Hydroxyphenyl)propanoyl]amino]-3-Methylbutanoyl]amino]propanoylamino]-4-oxobutanoic acid	HMDB0247776	0.51	0.48
2-Hydroxy-3-(4-methoxyphenyl) propanoic acid	HMDB0039427	0.51	0.45
2-Aminonicotinic acid	HMDB0061680	0.70	0.68
6-Amino-1-benzyl-5-methylamino-1H-pyrimidine-2,4-dione	HMDB0258564	0.72	0.66
Trigonelline	HMDB0000875	0.55	0.36
Fulvic acid	HMDB0252514	0.49	0.42
L-Tyrosine	HMDB0000158	0.71	0.65
7-hydroxy-3-(hydroxymethyl)-octahydropyrrolo[1,2-a]pyrazine-1,4-dione	HMDB0253728	0.71	0.65
Secologanin	HMDB0304482	0.48	0.38
Methyl dihydrophaseate	HMDB0039376	0.70	0.64

^a^ The differential metabolites were filtered by using *p* < 0.05 and FC > 1.20 or <0.83 as the criteria; ^b^ FC, fold change.

## Data Availability

The raw data supporting the conclusions of this article will be made available by the authors on request.

## Supplementary Materials

The following supporting information can be downloaded at: https://www.mdpi.com/article/10.3390/toxics12060389/s1, Figure S1: Fitted viability curves and calculated IC_50_ values of human liver cell lines exposed to 6PPD-Q for 48 h; Figure S2: Transformation products detected in cell culture media exposed to 6PPD-Q and the predicted potential transformation pathways based on chemical structure and known enzymatic reactions; Figure S3: LC-MS spectra for the identification of the phase I metabolite OH-6PPD-Q; Figure S4: LC-MS spectra for the identification of the phase II metabolite 6PPD-Q-O-Gluc; Table S1: Instrumental method for metabolomic analysis by UHPLC-Orbitrap MS; Table S2: Primer sequences of the oxidative-stress related genes for RT-qPCR; Table S3: Abbreviation, structure, molecular weight (MW), and transformation pathway of 6PPD-Q metabolic transformation products.
