# Supplementary material for: Effects of 6PPD-Quinone on Human Liver Cell Lines as Revealed with Cell Viability Assay and Metabolomics Analysis
Source: Toxics. 2024 May 26;12(6):389. doi: 10.3390/toxics12060389 (PMC11209231; doi:10.3390/toxics12060389)
Supplement: Supplementary file 1 [file toxics-12-00389-s001.zip › toxics-3007700-supplementary.pdf]

Supporting Information for

Effects of 6PPD-quinone on human liver cell lines as revealed by cell viability assay and metabolomics analysis

Yunqing Qi, Aiqing Qiu, Xinyue Wei, Yiting Huang, Qing Huang, Wei Huang*

College of Environment and Climate, Guangdong Key Laboratory of Environmental Pollution and Health, Jinan University, Guangzhou, Guangdong, China

Address correspondence to Wei Huang. Email: huangwei@jnu.edu.cn

The authors declare they have no actual or potential competing financial interest.

Table S1. Instrumental method for metabolomic analysis by UHPLC-Orbitrap MS.

Instrument	Thermo Fisher Scientific Vanquish UHPLC coupled to ThermoFisher Orbitrap 240 Mass Spectrometer		
Analytical Column	Waters ACQUITY UPLC®HSS T3 column (1.8μm 2.1*100mm)		
Column Temperature	40 ℃		
Mobile Phases	POS: (A) water with 0.1% formic acid, (B) acetonitrile with 0.1% formic acid<br>NEG: (A) water with 2 mM ammonium bicarbonate, (B) acetonitrile		
Gradient Profile	Time (min)	Percentage B (%)	Flow Rate (mL/min)
	0	2	0.35
	1	2	0.35
	3	50	0.35
	9	100	0.35
	12	100	0.35
	12.1	2	0.35
	15	2	0.35
Injection Volume	5 μL		
Type of ion source	H-ESI		
MS Parameters	Spray voltage (kV): 3500 V (POS), 2500 V (NEG)		
	Sheath gas (arbitrary units): 40		
	Auxiliary gas (arbitrary units): 10		
	Sweep gas (arbitrary units):1		
	Ion transfer tube temperature (°C): 325		
	Vaporizer temperature (°C): 350		
	AGC target: standard		
	Orbitrap resolution: 120,000		
	RF lens (%): 70		
	Scan range (m/z): 100-1000		
	Maximum injection time mode: Auto		
	Spectrum data type: profile		
MS/MS Parameters	Activation type: HCD		
	HCD collision energy (%): 30		
	±HCD collision energy (%): 5		
	Orbitrap resolution: 15000		
	number of dependents scans: 20		
	Isolation window (m/z): 2		
	AGC target: standard		
	Maximum injection time mode: Auto		
	Spectrum data type: profile		
	dynamic exclusion: Auto		

Table S2. Primer sequences of the oxidative-stress related genes for RT-qPCR.

Gene[a]	Gene-specific primer （5′–3′）	Primer Length
GADPH-F	ATGTCAAACATTGGTATCAATGGATTTGG	22
GADPH-R	TTTAATCCTTAGATTGCATGTACTTGAT	22
HO-1-F	AAGACTGCGTTCCTGCTCAAC	21
HO-1-R	AAAGCCCTACAGCAACTGTCG	21
CAT-F	TGGAGCTGGTAACCCAGTAGG	23
CAT-R	CCTTTGCCTTGGAGTATTTGGTA	23
SOD1-F	GGTGGGCCAAAGGATGAAGAG	21
SOD1-R	CCACAAGCCAAACGACTTCC	20
TrxR1-F	ATATGGCAAGAAGGTGATGGTCC	23
TrxR1-R	GGGCTTGTCCTAACAAAGCTG	21
GPx1-F	GTCGGTGTATGCCTTCTCGG	20
GPx1-R	CAGCTCGTTCATCTGGGTGT	20
Nrf2-F	GCAATGAAGACTGGGCTCTC	20
Nrf2-R	AAACCAGTGGATCTGCCAAC	20

[a] -F, forward primer; -R, reverse primer.

Table S3. Abbreviation, structure, molecular weight (MW), and transformation pathway of 6PPD-Q metabolic transformation products.

Abbreviation	Full name	Molecular formula	MW	Enzymatic reaction
OH-6PPD-Q	Mono hydroxy-6PPD-Q	$C_{18}H_{22}N_2O_3$	314.1630	Phase I
6PPD-Q-O-Gluc	6PPD-Q-O-glucuronide	$C_{24}H_{30}N_2O_9$	490.1951	Phase I and phase II

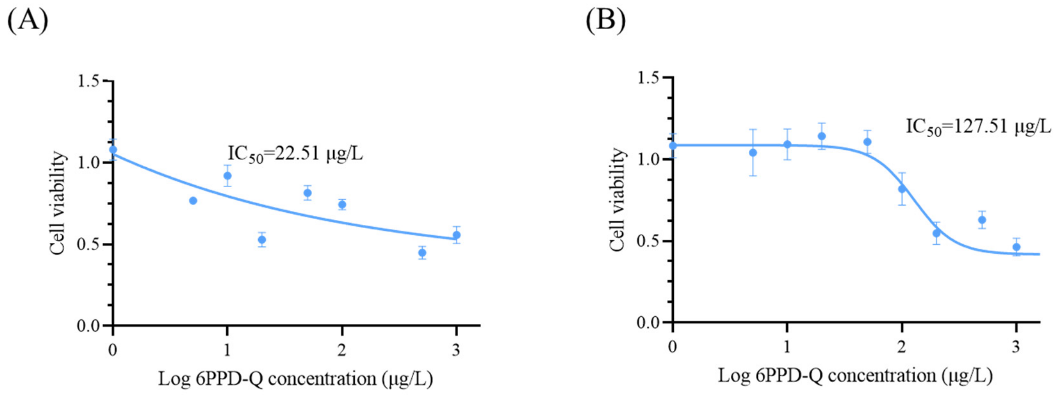

Figure S1. Fitted viability curves and calculated IC_{50} values of human liver cell lines exposed to 6PPD-Q for 48 h. (A) L02 cells; (B) HepG2 cells. Values shown are the mean and the error bars represent the standard deviation (n = 6 per group). The experiments were repeated three times. The IC_{50} values of 6PPD-Q to the cells were determined using nonlinear regression in GraphPad Prism software (GraphPad Software, San Diego, California USA).

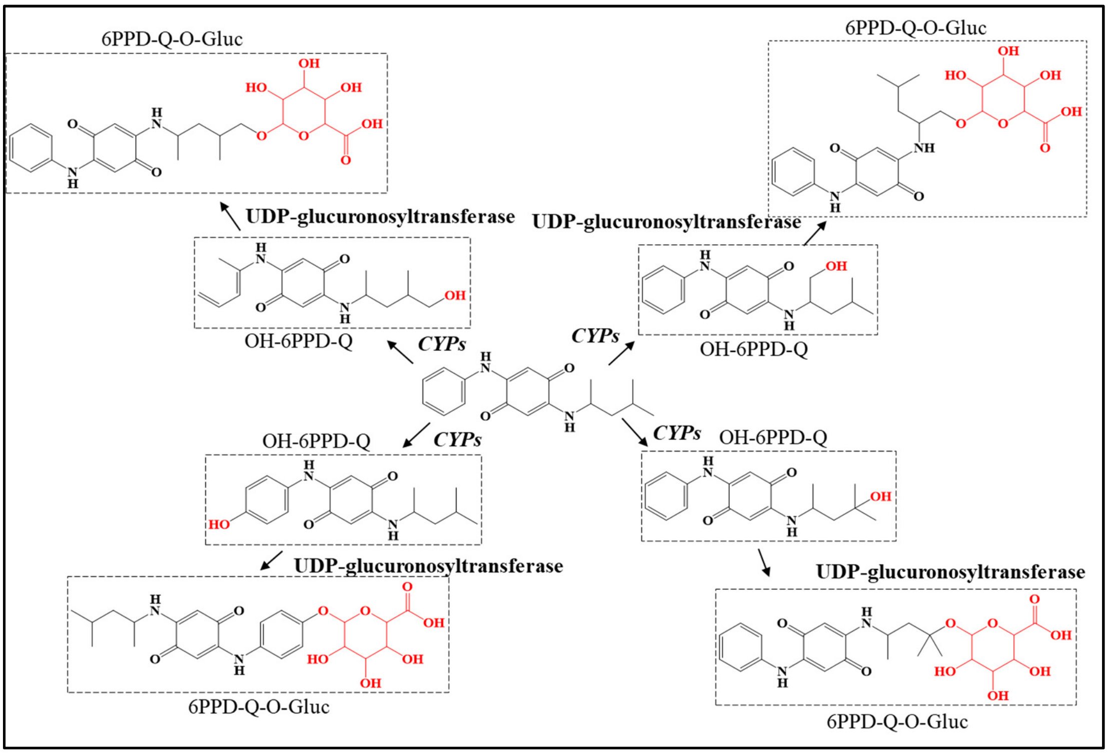

Figure S2. Transformation products detected in cell culture media exposed to 6PPD-Q and the predicted potential transformation pathways based on chemical structure and known enzymatic reactions. Arrows indicate enzymes that may be involved but do not indicate the order of the reaction. CYPs, cytochrome P450; NAT, N-acetyltransferase; UGT, UDP-glucuronosyltransferase. The phase I metabolizing enzyme CYP converts 6PPD-Q to OH-6PPD-Q, and the phase II metabolizing enzyme UDP-glucuronosyltransferase then converts OH-6PPD-Q to 6PPD-Q-O-Gluc.

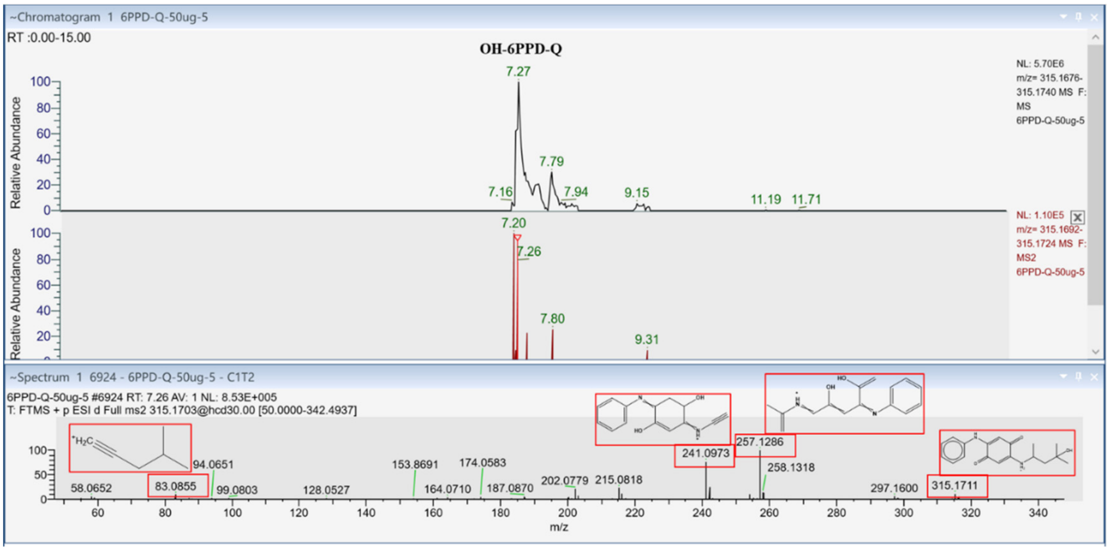

Figure S3. LC-MS spectra for the identification of the phase I metabolite OH-6PPD-Q.

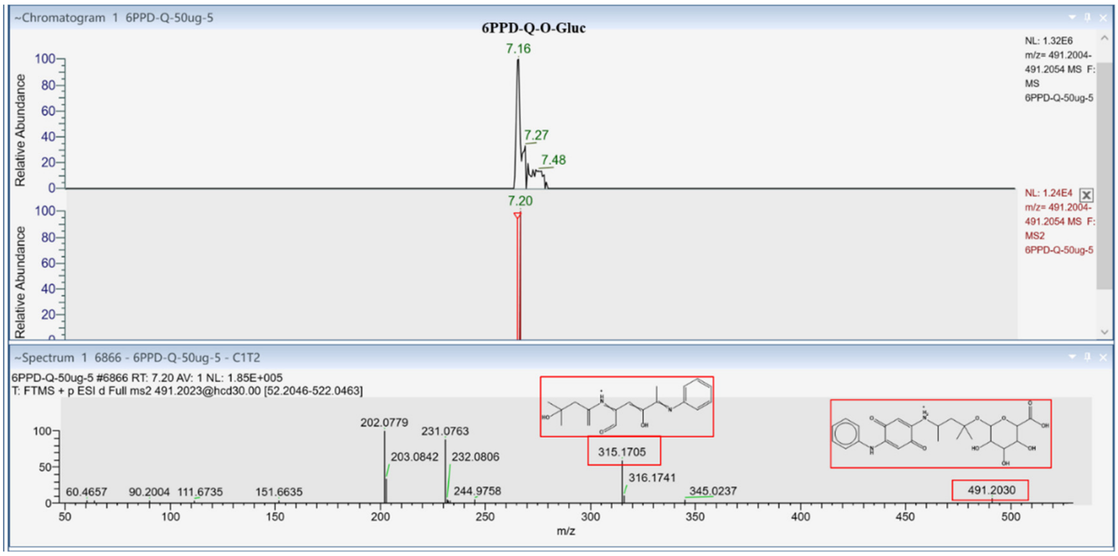

Figure S4. LC-MS spectra for the identification of the phase II metabolite 6PPD-Q-O-Gluc.
